# Avian Pathogens: Editorial and the Perspectives of Research

**DOI:** 10.3390/microorganisms10030543

**Published:** 2022-02-28

**Authors:** Kirill Sharshov

**Affiliations:** Laboratory of Molecular Epidemiology and Biodiversity of Viruses, Federal Research Center of Fundamental and Translational Medicine, 630117 Novosibirsk, Russia; sharshov@yandex.ru

## 1. Introduction

In the last ten years, humanity has faced new challenges in the field of human and animal health, including emerging viral infections. The widespread occurrence of emerging and re-emerging pathogens (e.g., highly pathogenic avian influenza virus (HPAIV), the new COVID-2019 of zoonotic nature, coronaviruses that cause severe acute respiratory syndrome (SARS), and Middle East respiratory syndrome (MERS)) presents a serious threat to humans. Avian pathogens cause devastating economic losses in poultry industries worldwide. Poultry is one of the main sources of protein in Asia. For instance, in China alone, with a population of more than 1.3 billion people, poultry meat consumption has increased to 19.6 kg/year per person (FAO). According to official estimates, this is over 25 million tons per year, or more than 12.7 billion birds. Therefore, poultry farming in Southeast Asia is considered one of the main “vessels” where avian pathogens can evolve. Wild bird migration makes the spread of these pathogens to other regions increasingly likely, as we have seen worldwide with the H5N8 influenza virus. In addition, avian pathogens can cause devastating epizootics among wild birds and threaten the planet’s bioresources. The importance of wild birds as potential vectors of disease has received recent renewed interest, especially in the frame of the global virome/microbiome concept. Understanding the spread of bacterial and viral pathogens in wild birds may serve as a useful model for examining the spread of other disease organisms, both among birds, and from birds to other taxa. This Special Issue aims to summarize recent advances and to attract new research articles that address existing pathogens in poultry and wild birds. It also aims to compare the epidemiological situation across avian pathogens or countries.

## 2. Results and Progress 

In this Special Issue, “Avian Pathogens”, we have received, reviewed and published a variety fof relevant studies on different existing topics. Three articles and one review were devoted to one of the most urgent global problems—avian influenza virus control. In a detailed review, Bisset et al. [1] analysed the potential of current highly pathogenic avian influenza (H7N7) virus in Australia for further influenza virus evolution and spread worldwide. Looking at the history of H7N7 in Australia, the authors consider in detail the possibility of increasing virulence and pathogenesis due to mutations, paying special attention to the Australian ostrich as a potentially unrecognized vessel for the mixing and reassortment of viruses. An interesting survey on the presence of viruses (mainly avian influenza and Newcastle disease viruses) in avifauna in Northern Italy and their public health and economic impact is provided by Trogu et al. [2]. They showed the presence of several viruses in different wild bird species from northern Italy, identifying that in most cases birds did not show clinical signs and could be active distributors of infection. Trovão et al. [3], from the USA, present the results of a multiyear surveillance of avian influenza virus in Duck Hunting Preserves (DHP), showing that such low viral gene flow demonstrated for DHPs confirms the hypothesis that mallards do not support long-term viral circulation, nor do they act as important sources of novel viruses in bird populations. Importantly, the authors suggest that such territories as regional sentinel stations, that reflect the viral diversity in local waterfowl populations, can provide an effective approach for long-term avian influenza surveillance. In studies concerning subtle mechanisms, Layton et al. [4] shed some light on interferon activity in chickens, which plays a major role inhibiting virus replication in DF1 Cells. Their findings support that the type I interferon receptor for the IFN signalling pathway is crucial for chickens and these results highlight the importance of interactions between avian and host pathogens and their innate immunity.

The second part of this Special Issue focuses on bacterial pathogens in veterinary medicine. Lozica and colleagues [5] provide a comprehensive genomic analysis of *Escherichia coli* isolated from Broilers after the administration of an autogenous vaccine. They sowed active circulation of the commensal bacterial strains, which can be a reservoir of resistance genes that are mostly associated with virulence genes. In a more practical sense, Hashish et al. [6] describe the development and validation of a novel TaqMan Real-Time PCR for the diagnosis of *Ornithobacterium rhinotracheale*. Such a newly developed assay is an important diagnostic tool for the sensitive and efficient diagnosis of ornithosis in clinical samples. Osuna-Mascaró et al. [7] provided genome-resolved metagenomic analyses and revealed the presence of a putative bacterial endosymbiont (*Rhinonyssidae*; *Mesostigmata*) in an avian nasal mite. Authors revealed and first described the presence of the endosymbiont, improving our overall understanding of the microbiota in these mites.

Another problem in veterinary medicine is the avian leukaemia virus. In this Special Issue, Kheimar et al. [8] showed that a genetically engineered chicken with original mutation of ALV-J receptor is resistant to Highly Pathogenic Avian Leukosis Virus Subgroup J, which can be useful for controlling this pathogen and improving poultry health in field conditions. 

Another interesting investigation was carried out on the study of parasites in wild birds. Golnar et al. [9] studied vector-borne blood parasites of the Great-Tailed Grackle (*Quiscalus mexicanus*) in East-Central Texas, USA, and demonstrated that this bird species can act as a very active natural reservoir for such blood parasites.

## 3. Perspectives of the “Avian Pathogens” Research 

A huge number of publications exist on the topic of avian pathogens. However, where are we now in this current direction and what current trends can be observed in this scientific and applied direction? Overall, the content of the Special Issue articles, in addition to the analysis of recent articles presented below, reveal the following current, hot, trends in avian pathogens.

One of the main trends in recent years has been the discovery of new viruses and the development of diagnostics. This is where the metagenomics approach comes to the rescue. Metagenomics make it possible to study in detail the diversity of communities, and therefore find out the mechanisms of their functioning and determine the metabolic relationships of various groups of living things. Undoubtedly, the main achievements in this area can be identified in research on human metagenomics (microbiome and virom of various organs and systems). However, metagenomic research is expanding, involving more and more taxa, including birds and their pathogens. In recent years, such new concepts and new directions in science, such as the “Global Microbiome” and “Global Virom”, have been formed [10,11]. Birds act as a significant gene source and amplify hosts in cross-species transmission and the emergence of novel viruses [12]. According to the latest data, even in Antarctica a large variety of potentially zoonotic viruses have been found [13].

In the last twenty years, we have observed the rapid development of viromics: different studies of viral abundance with very active sequencing of novel virus genomes worldwide. However, there is still a need to clearly identify the gaps in our modern understanding of wild bird and poultry viromes. Avian viromes can generate additional information to routine monitoring programs and clarify our understanding of how zoonotic viruses effect human health [14,15]. 

Currently, with the widespread development of technologies for next generation sequencing (NGS) and the analysis of metagenomic data, more and more information on microbial communities is becoming available to researchers. However, there are negligible works investigating both the microbiome and the virome of hosts. This is one of the main gaps identified in the analysis of current problems in this field. Additionally, the limitations of these numerous and interesting studies are that the biological properties and functions of many pathogens (viruses and bacteria) detected by sequencing remain unclear, since they cannot be cultivated and their life cycle cannot be studied directly. This problem is yet to be solved.

One of the most relevant research areas for agriculture, veterinary medicine and the protection of wild birds continues to be well-known pathogens; for example, the ongoing panzootia of highly pathogenic avian influenza (HPAI). Significant attention is paid to this subject in our Special Issue. Since 2017, the largest distribution of H5N8 with several waves has been registered in countries of Eurasia and Africa [16,17,18]. The recent detection of highly pathogenic avian influenza (H5N1) in Canada: Newfoundland and Labrador 2021 and Nova Scotia 2022, was a logical consequence. The detection of such a spread of HPAI viruses into the New World raises concerns of further virus dissemination across North and South America by wild bird migration [19].

The transmission of zoonotic pathogens from avian reservoirs to humans, such as avian influenza viruses, can result in severe disease cases and lead to a pandemic. On the other hand, human-adapted avian pathogens have been shown to have the potential to spread back to livestock, resulting in so-called “reverse zoonotic transmission”. Thus, it is important to control the circulation of pathogens within these avian reservoirs to decrease zoonotic transmission, for example with the development an efficient vaccination approach. It is also crucial to provide systematic surveillance of the various human-made avian reservoirs to provide a risk assessment for novel genetic pathogenic variants to emerge [20]. In recent decades, contamination of the environment with various pathogens has increased due to the intensity of animal farming, followed by the emergence of novel pathogens with pathogenic potential and a threat to global health [21]. 

In this regard, attempts to develop and improve new vaccines will not be stopped, and this will continue to be a trend in the future. The development of vaccination technologies has led to a significant increase in the number of poultry, but, in general, it has again led to a weakening of food security in the world. The problem of enhancement in vectors and pathogens and novel diseases in birds has led to the urgent need for transmission control for emerging infectious diseases and the study of animal–human interaction in terms of Global Health. One of the major directions is the development of new vaccine technologies, especially for global poultry farming Thomas et al. [22], summarizing these perspectives, describe the different challenges to be considered in the development of vaccines for animals. As humans and animals share a common environment and their health is closely connected, veterinary and medical services should actively cooperate to prevent risks to public health and work together in accordance with a One Health conception [23]. 

The development of new methods for rapid diagnosis of avian pathogens does not stop either. Of course, PCR remains the main approach. At the moment, scientists are discovering and describing more and more new viruses, owing to the accessibility of nucleic acid screening methods such as polymerase chain reaction (PCR), quantitative (real-time) polymerase chain reaction (qPCR) and reverse-transcription qPCR (RT-qPCR). The review from Padzil et al. [24] describes past results on and future trends of major technologies and their principles, benefits and limitations as promising point-of-care diagnostic methodologies against avian viruses. 

One of the new trends is an alternative to antibiotics in poultry farming: probiotics and the use of bacteriophages. Antibiotics used to be supplemented to animal feeds; however, they also kill bacteria that may be beneficial and enhance antibiotic-resistance in pathogenic bacteria. The global increase in multidrug-resistant infections caused by various pathogenic strains has raised concerns in veterinary and human medicine. To overcome the above problems, scientists are taking a great deal of measures to develop alternatives for antibiotics. Probiotics establish cross-feeding between different bacterial strains of the gut ecosystem and reduce blood cholesterol levels via bile salt hydrolase activity; beneficial effects of probiotics can be a potential alternative strategy to replace antibiotics in poultry [25]. Another perspective—bacterial viruses and bacteriophages—can be used therapeutically and could help us to alleviate the “antibiotic crisis” [26]. Actually, bacteriophages can successfully be used in the food industry in terms of quality control efforts against the microorganisms of birds such as Salmonella, Escherichia coli, Listeria, Campylobacter and others. Future advances will offer potential alternative methods of using bacteriophages to fight infections caused by multidrug-resistant bacteria [27]. For example, Campylobacter phages application to poultry carcasses has been suggested as a strategy to decrease Campylobacter concentrations during poultry processing. [28].

## 4. Conclusions

Obviously, the widespread occurrence of emerging and re-emerging pathogens presents a serious threat to humans. The importance of avian pathogen study has grown significantly since humans realized that avian diseases can have serious implications on different areas of life.

It was the aim of this Special Issue, “Avian Pathogens”, to shed some light on the different aspects of avian pathogens, focusing on their impacts on human health. Articles included covered a wide range of subjects within applied research, including avian influenza control, veterinary microbiology, the problem of antibiotic resistance, and the diagnostics of pathogens. The authors believe that with the rapid advancement of diagnostics and metagenomics studies, communities will achieve progress in the control and management of avian pathogens, which will impact positively on Global Health.

Overall, I would like to thank all the researchers who contributed to this Special Issue and whose mentioned articles were used to analyse the various perspectives of research. Since we are constantly accepting new articles, I would like to continue this Special Issue in the next edition on the same topics, in which I hope to focus more on other emerging viruses, virome and microbiome studies of wild birds and poultry and widespread veterinary problems that can have significant implications for global health.
